# Psychometric evaluation of the Major Depression Inventory among young people living in Coastal Kenya

**DOI:** 10.12688/wellcomeopenres.12620.1

**Published:** 2017-11-29

**Authors:** Mark Otiende, Amina Abubakar, George Mochamah, David Walumbe, Christopher Nyundo, Aoife M Doyle, David A Ross, Charles R Newton, Evasius Bauni

**Affiliations:** 1 Centre for Geographic Medicine (Coast), Kenya Medical Research Institute, Kilifi, Kenya; 2 INDEPTH (International Network for field sites with continuous Demographic Evaluation of Populations and Their Health in developing countries) , Accra, Ghana; 3 Pwani University, Kilifi, Kenya; 4 University Department of Psychiatry, University of Oxford, Oxford, UK; 5 London School of Hygiene & Tropical Medicine, London, UK

**Keywords:** Major Depression Inventory, youth, Kenya, psychometric evaluation

## Abstract

**Background**: The lack of reliable, valid and adequately standardized measures of mental illnesses in sub-Saharan Africa is a key challenge for epidemiological studies on mental health.  We evaluated the psychometric properties and feasibility of using a computerized version of the Major Depression Inventory (MDI) in an epidemiological study in rural Kenya.

**Methods**: We surveyed 1496 participants aged 13-24 years in Kilifi County, on the Kenyan coast. The MDI was administered using a computer-assisted system, available in three languages. Internal consistency was evaluated using both Cronbach’s alpha and the Omega Coefficient. Confirmatory factor analysis was performed to evaluate the factorial structure of the MDI.

**Results**:  Internal consistency using both Cronbach’s Alpha (α= 0.83) and the Omega Coefficient (0.82; 95% confidence interval 0.81- 0.83) was above acceptable thresholds. Confirmatory factor analysis indicated a good fit of the data to a unidimensional model of MDI (χ ^2^ (33, *N* = 1409) = 178.52 *p *< 0.001, TLI = 0.947, CFI = 0.961, and Root Mean Square Error of Approximation, RMSEA = .056), and this was confirmed using Item Response Models (Loevinger’s H coefficient 0.38) that proved the MDI was a unidimensional scale. Equivalence evaluation indicated invariance across sex and age groups. In our population, 3.6% of the youth presented with scores suggesting major depression using the ICD-10 scoring algorithm, and 8.7% presented with total scores indicating presence of depression (mild, moderate or severe).  Females and older youth were at the highest risk of depression.

**Conclusions**: The MDI has good psychometric properties.  Given its brevity, relative ease of usage and ability to identify at-risk youth, it may be useful for epidemiological studies of depression in Africa.  Studies to establish clinical thresholds for depression are recommended. The high prevalence of depressive symptoms suggests that depression may be an important public health problem in this population group.

## Introduction

Young people aged 10–24 years constitute about a third of the global population, and behaviour and mental health at this age have substantial health effects later in life ( Gore *et al.*, 2011). For instance, half of all adult mental health disorders first appear during the adolescent years ( Gore *et al.*, 2011), though many are undiagnosed and untreated prior to adulthood. Mental health disorders are common and are a leading cause of disability in young people globally ( Gore *et al.*, 2011; World Health Organization, 2014). In Africa Disability-Adjusted-Life-Years (DALYs) lost due to depressive disorders is higher than in other regions and neuropsychiatric disorders are estimated to contribute 5% of the total DALYs lost ( Vos *et al.*, 2016; World Health Organization, 2008). Studies in Kenya have suggested that roughly one-quarter of out-patients attending health facilities have mental disorders ( Kiima *et al.*, 2004). Despite this, prevention, screening, and treatment of mental health problems and neuropsychiatric disorders have been widely overlooked in public health programmes in low and middle-income countries (LMICs).

The lack of adequately standardized and psychometrically robust measures for evaluating mental health problems ( Mutumba *et al.*, 2014; Patel *et al.*, 2007; Saxena *et al.*, 2007; Tennyson *et al.*, 2016) has contributed to the shortage in high quality epidemiological studies to estimate the burden of mental health problems in Africa. Only recently have there been efforts, to develop, adapt and evaluate measures of mental health ( Betancourt *et al.*, 2009; Mels *et al.*, 2010) for use in Africa. However, there are still very few reports of the psychometric properties of the widely used measures of mental health ( Mutumba *et al.*, 2014).

Several screening tools for depression have been used in previous studies in Kenya, including the Patient Health Questionnaire (PHQ-9), the Center for Epidemiologic Studies-Depression (20-item CES-D) questionnaire, and Beck’s Depression Inventory (21-item BDI) ( Kingori *et al.*, 2015; Seth *et al.*, 2014). Although an extensive study in Kilifi found that the BDI was a good measure of depressive symptoms, it is lengthy and therefore not suitable for use in large epidemiological studies ( Abubakar *et al.*, 2016). Similarly, the CES-D has 20 items, thus likely to present the same problems as the BDI. PHQ-9 is constructed to screen for major depression, but does not measure depression severity ( Bech *et al.*, 2015). We chose to evaluate the psychometric properties of the Major Depression Inventory (MDI) ( Bech *et al.*, 2001) when applied to young people in a rural African context, because of its brevity and validity for assessing common depressive disorders ( Konstantinidis *et al.*, 2011). Compared to other scales that measure depressive disorders, the MDI was shown to be superior to the Zung-Self-Rating Depression Scale (Zung-SDS) and BDI ( Bech *et al.*, 2015; Konstantinidis *et al.*, 2011).

The selection of measures for adaptation and evaluation for use in a new context requires careful consideration of a number of factors. First, the validity of the measure in the original context and in other cultural contexts where it was validated should be considered. The MDI is a measure which has accrued a significant amount of evidence on its construct validity, and has sound psychometric properties both in its original sample ( Olsen *et al.*, 2003) and across different cultural contexts such as Greece ( Fountoulakis *et al.*, 2003), Brazil ( Parcias *et al.*, 2011) and Egypt ( Fawzi *et al.*, 2012). Second, it is important to consider the feasibility of using the measures in the new context. The brevity and relative ease of use of the MDI makes it particularly suitable for screening depressive symptoms in large epidemiological studies compared to other scales or questionnaires. Lastly, the cultural appropriateness and relevance of the items for the new context is critical for the validity of measures being moved from one cultural context to the other. Thus, content validity of the items should be ascertained to ensure that items are suitable for use in the new context. In Kilifi an earlier qualitative study of symptoms closely associated with depression identified an overlap between the themes, symptoms and contents of the MDI with community perceptions of depressive symptoms ( Abubakar *et al.*, 2016). For instance, some of the key symptoms identified by the members of the community included feelings of sadness, loss of weight, and lack of sleep among others. The information collected from our qualitative work provides a good indication that the items covered by MDI were relevant to rural Kenya. These considerations favored the selection of MDI as the most suitable measure of depressive symptoms to evaluate for use in this context.

In addition, social desirability bias is a common problem inherent in face-to-face interviews that involve sensitive questions. Social desirability is the inclination to report behaviors that are viewed in a favorable way by society ( Beauclair *et al.*, 2013). In order to mitigate against this form of bias and other concerns of privacy when responding to sensitive questions, appropriate interviewing/data collection mechanisms, such as the Audio Computer-Assisted Self-Interviewing (ACASI) system need to be utilized ( West and Peytcheva, 2014). In this study, interviews were conducted using the ACASI in three different languages.

When scales and measures are used in another context, it is important to evaluate their psychometric properties, as it cannot be assumed that psychometric properties from the original contexts are retained. An adequate psychometric evaluation process needs to check for reliability, validity and feasibility of the scale.

Consequently, we performed an evaluation of MDI and evaluated the feasibility of using this tool in rural Coastal Kenya. Specifically, we aimed to evaluate:

a) the internal consistency of the MDI;

b) if the MDI would present with the single factorial structure as envisioned by the authors;

c) whether there is equivalence of these structures across sex and age.

### Ethical statement

The Kenya Medical Research Institute National Scientific and Ethical Committee approved this study (approval number: SSC 2823). Written informed consent was obtained from all study participants prior to participation. For participants aged less than 18 years, parental consent and the participant’s assent were obtained.

## Methods

### Study site

This study was carried out from August to December 2014 and utilized survey data collected from the Kilifi Health and Demographic Surveillance System (KHDSS) in coastal Kenya. This was part of a multi-site survey on health behaviours of young people (13–24 years) conducted in two INDEPTH network sites between August to December 2014, the other one being the Dodowa Health and Demographic Surveillance System in Ghana.

Within the KHDSS, births, deaths, and migration of individuals are recorded routinely after every four months ( Scott *et al.*, 2012). Geographically, the KHDSS covers an area of 891 km ^2 ^and has a residential population of 280,000 of which young people constitute approximately a quarter. Almost 71% of the Kilifi County population lives below the poverty line [a monthly income of less than $18 and $33 for rural and urban dwellers, respectively] ( Commission of Revenue Allocation, 2011). Only 13% of Kilifi County residents have secondary level of education or above ( KNBS, 2013) and therefore a significant number of adults are unable to adequately participate in written interviews. The majority of the population in Kilifi belong to the Mijikenda ethnic group who mainly speak two Bantu languages; Kigiryama (the local vernacular) and Kiswahili (the lingua franca and the national language). Within the KHDSS area there is one county hospital (Kilifi County Hospital) and more than 39 health centres and dispensaries spread across the area ( Scott *et al.*, 2012). Within Kilifi County Hospital, there is one specialist psychiatric clinic, which is run by a specialized psychiatric nurse and serves up to 70 patients a day. The common problems presented are schizophrenia/psychosis, seizure disorders followed by depression and other mood disorders ( Bitta *et al.*, 2017). There were no formal measures of evaluation and diagnosis of depression at the time of this study.

### Sampling procedures and sample size

A representative random sample of young people aged 13 to 24 years residing in the KHDSS was recruited. A resident was defined as a person who had spent at least one night in a household in the KHDSS area and had stayed or intended to stay for at least three months ( Scott *et al.*, 2012). To calculate the required sample size, we assumed a prevalence of 0.5 for all factors being measured in the study to obtain the largest possible sample size. With a precision (half-width) of 0.0250 and a two-sided 95% confidence interval, a sample size of 1514 was derived from a population of 100,000. We further adjusted for a non-response rate of 10%, which gave a final sample size of 1,665.

### Socio-demographic, anthropometric and behavioral measures

Questions on socio-demographic, anthropometric measures and behavioral patterns selected from the Global School-based Health Survey (GSHS) were administered with the MDI.

 The major depression inventory (MDI) is a self-reported measure of depressive symptoms with items examining the moods one experienced in the previous 2 weeks. The measure covers DSM-IV and has 12 items, though only 10 items are scored, since items 8 (a and b) and items 10 (a and b) are collated into two items. The questions are scored on a 6 point Likert scale with ‘0’ being at no time and at ‘5’ all the time. The individual items measure how much of the times the symptoms have been present during the past 14 days. According to Olsen (2003) the MDI can be used both as:

- A diagnostic instrument with the algorithms leading to the DSM-IV or ICD-10 “major” or “moderate to severe depression”
- A depression rating scale to measure depression severity in which the total score of the 10 items is a sufficient statistic.

In this study, we assessed depressive symptoms using both methods. We categorized the severity of depression following the MDI scores: 20–24 for mild depression; 25–29 for moderated depression; and 30 or higher for severe depression.

The MDI, which originally is developed and evaluated in a Danish population, has been found to have high sensitivity (between 0.86 and 0.92) and specificity (between 0.82 and 0.86) compared to Schedules for Clinical Assessment in Neuropsychiatry (SCAN) as the index of validity ( Bech *et al.*, 2001). Likewise, in the original population its internal and external validity was evaluated using both classical methods (i.e. Cronbach’s Alpha) and modern methods (i.e. Mokken scale analysis) and the MDI was found to be a unidimensional scale ( Olsen *et al.*, 2003). Consequently, in this study, we use both the classical and modern methods to evaluate the validity of the MDI in our context.

### Translation of questionnaire items

All the items (including the MDI items) were translated to Swahili and Giryama. Forward translation of the MDI to English and Giryama was done by one of the investigators who is conversant with all these three languages and back translation was done by other independent translators. Through a harmonization meeting, it was ensured that all the translated items of the MDI were equivalent to the original English version in terms of meaning and context.

### Measure of behavioral patterns among the youth

Of interest to this study were measures of patterns of alcohol use and experiences of bullying which were part of behavioral data collected during the survey. Drinkers were defined as those who consumed any amount of alcohol. Bullying was classified into three categories: those who were never bullied; those who were less bullied (2 or 3 times); and those who were often bullied (4 to 7 times). The period of assessment was within 30 days before the day of the interview.

### Data collection procedures

The interviews were done using an Audio Computer-Assisted Self–Interview software (ACASI) to ensure respondent privacy and improve the accuracy of the information collected. A previous study demonstrated that participants found the ACASI modality to be acceptable, private and user friendly ( Beauclair *et al.*, 2013). All questions were recorded and programmed into the ACASI software in three languages (English, Swahili and Giryama). Data was collected during household visits where study participants were instructed in detail on how to use the ACASI prior to being given a laptop and a pair of headphones. Participants were left to complete the interview in private.

Prior to the start of the survey, a pilot was done to assess the functionality of the ACASI software. This pilot was covered by ethical approval that was granted for the study. During the pilot, respondents also filled out physical paper forms (alongside ACASI), and these responses were later compared to the responses from ACASI. For each question, response from the paper questionnaire and ACASI version were compared and were considered similar if they were exactly the same.

### Statistical analyses

***Internal consistency.*** We computed the Cronbach’s alpha and Omega coefficient with 95% confidence interval to assess the internal consistency of the scale. The omega coefficients were computed using the MBESS package in R ( Kelley, 2007). Owing to criticisms, surrounding the use of Cronbach’s alpha in the assessment of reliability of psychological scales ( Black *et al.*, 2014; Dunn *et al.*, 2014; Iacobucci & Duhachek, 2003), we also computed the Omega coefficient. Conventionally, both coefficients are acceptable when the value is above 0.70 ( Cicchetti, 1994).

***Factorial structure.*** The factorial structure of the MDI was assessed using both classical methods (Exploratory Factor Analysis) and modern methods (Item Response theory). We performed factor analysis using iterated principal factors. A scree-plot and eigenvalues of all possible factors were evaluated to determine the number of factors to retain. Any factor with an eigenvalue greater than 1 was retained. Mokken scale analysis, a class of statistical models called item response models (IRM), was also used to assess whether the MDI was a unidimensional scale. A Loevinger’s H coefficient between 0.30 and 0.39 was considered an acceptable indication of a unidimensional scale ( Mokken, 1971; Olsen *et al.*, 2003)

To assess the equivalence of psychological measures across age and sex, we used a multi-group confirmatory factor analyses (MGCFA). A unidimensional model including all 10 items was estimated. We assessed the goodness of fit for each model using Chi-Square, the Tucker-Lewis Index (TLI), and the Comparative Fit Index (CFI). The general guideline for TLI and CFI is that values greater than 0.95 are considered to reflect an excellent fit, while those between 0.95 and 0.90 are considered indicative of an acceptable fit. We report the Root Mean Square of Approximation (RMSEA), which is sensitive to model misspecification: values of less than 0.06 are considered, indicative of a good fit while those between 0.06 and 0.08 are considered indicative of an acceptable model.

In a multi-group analysis, the change in CFI is an important indicator for assessing the suitability of hierarchically nested models. Thus, a CFI change of less than 0.01 is taken to be supportive of the more restrictive model ( Milfont & Fischer, 2010; van de Schoot *et al.*, 2012). It is recommended that three levels of statistical equivalence are evaluated ( van de Schoot *et al.*, 2012) i.e. configural equivalence, metric equivalence and scalar invariance. Configural equivalence is achieved when items in the measuring instrument show the same pattern of factor loadings within each group. Metric equivalence (second level) indicates whether or not respondents from different groups respond to the questions in a similar manner. It requires that the factor loadings linking items and a construct are equal, and is an indicator of similarity of measurement unit (the scale metric). Scalar invariance (third level), which requires equality in both factor loading and intercepts across groups. Mean score comparisons are only permissible when one achieves scalar (full or partial) invariance and metric (full or partial) invariance then. Only then is it permissible to compare the relationship between variables across groups ( Milfont & Fischer, 2010).

All analysis except for Omega coefficients were done using STATA version 13.1 (StataCorp, College Station, Tex).

## Results

Complete data for this analysis was obtained from 1496 participants, whose characteristics are shown in Table 1. Only 2% could not work with ACASI and therefore were assisted in completing the interviews. All of the respondents had some form of education; 62% had reached or were still in primary school, while 3% had reached or were still in tertiary institutions. Overall, 11% admitted to have taken alcohol within 30 days prior to the interview and 25% reported being bullied at least once within 30 days preceding the interview.

**Table 1.** Socio-demographic characteristics of respondents.

	Total Sample	13–14 yrs	15–19 yrs	20–24 yrs
	(N=1496)	(n=351)	(n=689)	(n=456)
Sex		n (%)	n (%)	n (%)
Male	773	187 (24.2)	359 (46.4)	227 (29.4)
Female	723	164 (22.7)	330 (45.6)	220 (31.7)
Education*				
Primary	921	320 (34.7)	506 (54.9)	95 (10.3)
Secondary	128	3 (2.3)	61 (47.7)	64 (50.0)
Tertiary	40	0 (0)	10 (25.0)	30 (75.0)
Other/None	310	26 (8.4)	96 (31.0)	188 (60.7)
Missing	97	2 (2.1)	16 (16.5)	79 (81.4)
Drinking habits*				
Non-drinkers	1233	327 (26.4)	578 (46.9)	328 (26.6)
Drinkers	169	9 (5.3)	62 (36.7)	98 (58.0)
Missing	94	15 (16.0)	49 (52.1)	30 (31.9)
History of bullying*				
Never bullied	1118	257 (23.0)	509 (45.5)	352 (31.5)
Less bullied	313	79 (25.2)	151 (48.2)	83 (26.5)
Often bullied	59	15 (25.4)	28 (47.5)	16 (27.1)
Missing	6	0 (0)	1 (16.7)	5 (83.3)

### Reliability

Cronbach’s alpha for MDI was 0.83, which is above the acceptable threshold score of 0.70. We also obtained an omega coefficient of 0.82 (95%CI 0.81-0.83) indicating that regardless of the method we used the internal consistency was acceptable for our sample. ( Table 2)

**Table 2.** Measure of internal consistency using Cronbach’s alpha and Omega coefficient.

	Mean MDI	Cronbach’s Alpha	Omega (95% CI)
Overall	7.25	0.83	0.82 (0.81, 0.83)
Sex			
Male	6.77	0.81	0.80 (0.79, 0.82)
Female	7.79	0.85	0.83 (0.82, 0.85)
Age			
13–14	5.43	0.84	0.83 (0.81, 0.86)
15–19	7.51	0.82	0.81 (0.79, 0.83)
20–24	8.42	0.84	0.82 (0.80, 0.85)
Language			
Swahili	7.22	0.84	0.82 (0.81, 0.84)
English	6.59	0.75	0.76 (0.62, 0.89)
Giriama	8.13	0.75	0.72 (0.61, 0.83)

### Factorial structure

To evaluate the factorial structure of the MDI, we only utilized data of those who completed the interview in Swahili (N=1386), since there were insufficient number of participants that completed the interview in English (n=57) and Giryama (n=53).

The results of the exploratory factor analysis showed a single factor that had an eigenvalue of 3.44 with the other factors having eigenvalues of less than 0.3. All the 10 items appeared to load well onto the single factor that had the highest eigenvalue. All the items with exception of item 7 (difficulty in concentrating) had Loevinger’s coefficient greater than 0.3. Table 3 shows the factor loadings and Loevinger’s coefficient of the 10 items of the MDI. Overall Loevinger’s coefficient for the 10 items was 0.38 indicating that the MDI is a unidimensional scale.

**Table 3.** Factor loadings from exploratory factor analysis (EFA) of the 10-item Major Depression Inventory.

Item	Factor 1	Loevinger’s coefficient of homogeneity
**1**	0.59	0.37
**2**	0.57	0.37
**3**	0.65	0.41
**4**	0.62	0.38
**5**	0.65	0.40
**6**	0.53	0.37
**7**	0.45	0.29
**8**	0.67	0.43
**9**	0.58	0.37
**10**	0.53	0.37
**All items**	Eigen value = 0.53	H ^s^=0.38

*H ^s –^ Loevinger’s scalability coefficient for MDI

Using confirmatory factor analytic approaches, we tested a single factor model as originally conceptualized by **t**he developers of the MDI in the original population. The data indicated that the model did have a good fit to the data at the configural level with the fit indices being within acceptable limits, χ ^2^ (35, *N* = 1386) = 271.023, *p* < 0.001, TLI =0 .916, CFI = 0.935, and RMSEA = 0.070 (fit indices prior to adding the correlated error). However on inspection of the modification indices, we found that for several items there was a large residual error, suggesting correlation between items. These items were correlated since theoretically, they may have a shared variance beyond that in the other items. The correlated items were 3 and 5 (lack of energy and feelings of guilt) and 2 and 8 (low interest in daily living activities and being restless or subdued). After modifying the model to improve its fit (i.e. by accounting for the correlation between the items 3 and 5), the model had a better fit to the data at the configural level with the fit indices in the modified model changing to: (χ ^2^ (33, *N* = 1386) = 173.221 *p* < 0.001, TLI = 0.947, CFI = 0.961, and RMSEA = 0.055).

### Invariance analysis

**Figure 1.**
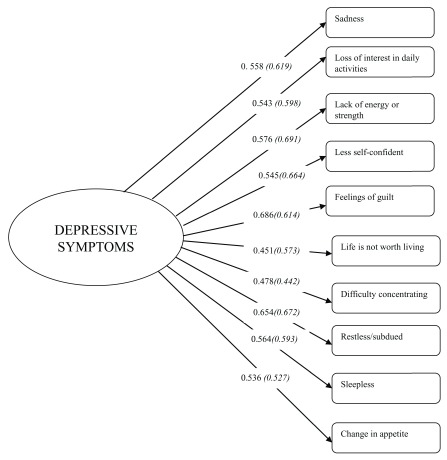
Factorial structure of the MDI items by sex. Italicized numbers in parentheses represents results from females.

Using MGCFA, we investigated the equivalence of the identified model across age and sex. Results indicated that the scale achieved scalar invariance across sex. The fit indices in this case were: χ ^2^ (84, *N* = 1386) = 295.799, *p* < 0.001, RMSEA = 0.060, TLI = 0.937, CFI = 0.941, and ∆CFI= 0.004 (see Table 4). Additionally, factor loadings for each item by sex were all significant ( Figure 1). Similarly, we investigated the equivalence of the identified model across age groups as previously defined. Scalar invariance was once again achieved and the fit indices were: χ ^2^ (135, *N* = 1,386) = 416.25, *p* < 0.001, TLI = 0.923, RMSEA = 0.067, CFI = 0.923, and ∆CF I= 0.008.

**Table 4.** Measurement Invariance.

	χ² (df)	∆χ² (∆ *df*)	RMSEA	TLI	CFI	∆CFI
Sex						
Configural	247.06 (66)		0.063	0.932	0.950	
Metric	272.65 (75)	25.58 (9)	0.062	0.934	0.945	0.005
Scalar	295.80 (84)	23.15 (9)	0.060	0.937	0.941	0.004
**Age**						
Configural	311.55 (99)		0.068	0.921	0.942	
Metric	367.54 (117)	55.99 (18)	0.068	0.921	0.931	0.011
Scalar	416.25 (135)	48.70 (18)	0.067	0.923	0.923	0.008

### Prevalence of major depressive symptoms

We observed that 3.6% [95%CI 2.7, 4.7] of our youth presented with DSM-IV major depressive symptoms. Female and older adolescents had higher prevalence of DSM-IV major depressive symptoms. No statistically significant difference in the prevalence of DSM-IV major depressive symptoms was observed across sex and age sub-groups.

We carried out additional analysis of the summed MDI scores and based on the conventional threshold score (i.e. higher than 20 indicating the presence of depression), we found that that 8.7% [95% CI 7.3, 10.2] of the youth had depressive symptoms. In this regard, the females had significantly higher prevalence (P-Value 0.0053) of 10.8% [95% CI 8.6, 13.3] compared to that of males at 6.7% [95%CI 5.1, 8.7]. The highest prevalence of depressive symptoms was observed among females aged 20–24 years (see Table 5).

**Table 5.** Mean scores and prevalence of major depressive symptoms. *: P<0.05, significant difference between two groups.

			Prevalence [95% confidence intervals]				
			MDI Score (CI)				DSM-IV % [CI]
		Mean MDI Score (CI)	MDI Score ≥ 20	Mild	Moderate	Severe	Symptoms of Major Depression
				(20–24)	(25–29)	(30 or more)	
All (n=1496)		7.3 [6.9, 7.6]	8.7% [7.3, 10.2]	4.3% [3.3, 5.4]	2.5% [1.7, 3.4]	1.9% [1.3, 2.8]	3.6% [2.7, 4.7]
Females (n=723)		7.8 * [7.2, 8.3]	10.8% * [8.6, 13.3]	5.0% [3.5, 6.8]	2.9% [1.8, 4.4]	2.9%* [1.8, 4.4]	4.4% [3.0, 6.2]
Males (n=773)		6.8 * [6.3, 7,2]	6.7% * [5.1, 8.7]	3.6% [2.4, 5.2]	2.1% [1.2, 3.3]	1.0% * [0.4, 2.0]	2.8% [1.8, 4.3]
Age 13–14 (n=351)	Both (351)	5.4 [4.8, 6.1]	6.0% [3.7, 9.0]	3.4% [1.8, 6.0]	0.8% [0.2, 2.5]	1.7% [0.6, 3.7]	2.6% [1.2, 4.8]
	Females (164)	5.9 [4.9, 7.2]	9.1% * [4.7, 13.6]	5.5% * [2.5, 10.2]	1.2% [0.1, 4.3]	2.4% [0.7, 6.1]	4.3% [1.7, 8.6]
	Males (187)	5.0 [4.2, 5.8]	3.2% * [0.7, 5.7]	1.6% * [0.3, 4.6]	0.5% [0, 3.0]	1.1% [0.1, 3.8]	1.1% [0.1, 3.8]
Age 15–19 (n=689)	Both (689)	7.5 [7.0, 8.0]	8.9% [6.8,11.2]	4.9% [3.4, 6.8]	2.2% [1.2, 3.6]	1.7% [0.9, 3.0]	3.0% [1.9, 4.6]
	Females (330)	7.8 [7.1, 8.6]	10.3% [7.0, 13.6]	4.8% [2.8, 7.8]	2.4% [1.1, 4.7]	3.0% * [1.5, 5.5]	3.3% [1.7, 5.9]
	Males (359)	7.2 [6.6, 7.9]	7.5% [4.8, 10.2]	5.0% [3.0, 7.8]	1.9% [0.8, 4.0]	0.6% * [0.1, 2.0]	2.8% [1.3, 5.1]
Age 20–24 (n-456)	Both (456)	8.4 [7.8, 9.1]	10.5% [7.9, 13.7]	3.9% [2.4, 6.2]	4.2% [2.5, 6.4]	2.4% [1.2, 4.3]	5.3% [3.4, 7.7]
	Females (229)	9.2 * [8.2, 10.2]	12.7% [8.4, 17.0]	4.8% [2.4, 8.4]	4.8% [2.4, 8.4]	3.1% [1.2, 6.2]	6.1% [3.4, 10.0]
	Males (227)	7.7 * [6.9, 8.6]	8.4% [4.8, 12.0]	3.1% [1.2, 6.3]	3.5% [1.5, 6.8]	1.8% [0.5, 4.5]	4.4% [2.1, 8.0]

## Discussion

We set out to evaluate the psychometric properties of the MDI. Our results indicate that the MDI in rural Kenya presents with acceptable internal consistency. Our results are supported by other studies that have evaluated the MDI in other geographical settings for example the Greek version (alpha of 0.86) ( Fountoulakis *et al.*, 2003) and the Arabic version which had an alpha of (0.91) ( Fawzi *et al.*, 2012). This possibly implies that the internal consistency of MDI is fairly stable across regions. Moreover, our high internal consistence obtained using the Cronbach’s alpha is further endorsed by the high Omega coefficient that we obtained.

Our findings from the exploratory factor analysis also indicate that MDI was measuring a single construct in our population. This conclusion was supported by the acceptable range of the Loevinger’s H coefficients ( Mokken, 1971; Olsen *et al.*, 2003) that we obtained when we utilized other modern psychometric methods besides exploratory factor analysis. 

We carried out a series of multi-group confirmatory factor analysis to evaluate the factorial structure and invariance of this structure across sex and age groups. Evaluating the invariance of a scale across different sub-samples is very important as studies indicate that when scales that are non-invariant are used to compare results there is a risk of reaching invalid conclusions ( Bontempo *et al.*, 2007; Chen, 2008). Our results indicate that MDI was not only unidimensional, but also that sub-groups i.e. males versus females and different age groups responded in a similar manner to the questions. The results add to the extant literature by showing that this measure, which is already widely used; can be applied across sub-samples in a statistically equivalent manner.

An important challenge in carrying out assessments in Africa is the multilingual nature of the population ( Foxcroft, 2004). In the Kenyan context for instance, an average youth may be able to speak three languages with different levels of fluency. Therefore, the question that arises is: which is the best language to use in the administration of the question? In our case we had three options, Swahili, Giryama and English, with participants choosing at the touch of a button, what language they would like to use at any point of the test. We had planned to evaluate the language equivalence of the measures to ensure that the use of different languages does not threaten validity, but most of our participants preferred to use the Swahili version making it difficult to evaluate the other language versions. Future work, will aim to target the evaluation of MDI in this other languages to provide us with information that allows us to utilize it with confidence.

We found the prevalence of DSM-IV depression and ICD-10 depression to be 3.6% and 8.7% respectively, which was consistent with the study conducted on the Danish general population which had a prevalence for DSM-IV depression and ICD-10 depression of 3.5% and 8.8% respectively for individuals aged 20–34 years. The main difference between the two countries is that Denmark is a high resource country compared to Kenya. This consistency gives further evidence of the robustness of the construct of depression in different settings ( Olsen *et al.*, 2004). We have shown that the MDI measures a single construct from evaluating its factorial structure and shown that the MDI is a unidimensional scale,using Item Response Models. These findings are consistent to the ones found in the original population from which the MDI was initially used.

As reported elsewhere, females in our sample reported higher prevalence of depressive symptoms and this seems to increase with age. In support of our findings, a recent study involving more than 10 European countries ( Boyd *et al.*, 2015) reported that “ *it has also been shown that females tend to report more depressive symptoms than males” (pg. 251) .* This is consistent with what has been reported in other parts of the world where females are usually at a higher risk of experiencing an episode of depression compared to males ( Mazur-Mosiewicz *et al.*, 2015; Pesola *et al.*, 2015). This difference can also be due to gender-biased items in the MDI. For instance, some items (fear, loneliness, crying, weight loss, fatigue, suicide ideation, appetite loss) in the Beck Depression Inventory are known to have a greater value for girls ( Salle *et al.*, 2011). Some of these items are also in the MDI.

An additional methodological issue we wanted to evaluate was the acceptability and feasibility of using a computer assisted assessment approaches. Due to low literacy levels, and a general lack of reading habits among members of our target population, using written questionnaires is not always possible in this context. We have previously used oral interviews when carrying out mental health, and other psychological assessment ( Abubakar *et al.*, 2016; Kariuki *et al.*, 2012). However, oral interviews may lead to response bias since interviewees may not always give the most truthful answer when dealing with highly personal and sensitive issues. It was therefore encouraging to observe that only a few (2%) of the participants needed assistance through the test administration procedures, which is an indication, that computer-assisted administration is an acceptable approach in our context. Given the many advantages such as increased anonymity and feelings of privacy especially when dealing with sensitive topics, it is encouraging to know that this is a method with potential for further development. After the pilot, before the main survey, on comparing responses from the paper questionnaires versus responses from ACASI, we found that the responses were 100% similar showing just how feasible ACASI is as a mode of interviewing persons aged 13–24 years in our setting.

Most mental disorders are often misdiagnosed because general health workers lack the diagnostic skills and training required to manage such cases ( Kiima *et al.*, 2004). Having a screening tool such as the MDI will reduce the cases of misdiagnosis and given that it is self-administered, and easy to understand it can easily be deployed even in health centers where trained psychiatrists are not available.

Since the last prevalence study on depressive disorders in Kenya was done in the 90s, it is important to report prevalence studies of depressive disorders even if they are from specific subsets of the population in order to increase the evidence base. We think that the MDI can be used in other rural parts of Kenya similar to the population in Kilifi to screen for depressive symptoms.

By focusing efforts towards screening for depressive symptoms in adolescents and youth and providing timely interventions, many of the cases of depressive disorder that are diagnosed in older ages can be reduced.

### Limitations

Our study has several strengths and adds salient information on the applicability of MDI in our context. Validity is a complex construct with various facets to it. We have been able to confirm structural validity, although we did not carry out clinical validation to evaluate the most suitable thresholds in this rural Kenyan population. This is essential to ensure that the current recommended approaches for estimating prevalence are valid for these settings. Future studies should focus on this, so as to further accumulate the evidence on the validity of the MDI. Future research should also examine test-re-test reliability and language invariance of the measure, particularly as the measure does not seem to have undergone cognitive pre-testing to ensure that the questions are understood as intended.

## Conclusions

Our results indicate that MDI has acceptable psychometric properties and is a suitable instrument for screening depressive symptoms in young people living in rural Kenya.

## Data availability

Data supporting the findings of this study are available from the KEMRI Institutional Data Access/Ethics committee, for researchers who meet the criteria for access to confidential data. Details of the criteria can be found in the KEMRI-Wellcome data sharing guidelines. Access to data is provided via the KEMRI-Wellcome Data Governance Committee: Data_Governance_Committee@kemri-wellcome.org; Tel, +254708587210; Contact person, Marianne Munene (Secretary; Tel, +254709983436).
