# Supplementary material for: Psychometric evaluation of the Major Depression Inventory among young people living in Coastal Kenya
Source: Wellcome Open Res. 2017 Nov 29;2:113. [Version 1] doi: 10.12688/wellcomeopenres.12620.1 (PMC5968359; doi:10.12688/wellcomeopenres.12620.1)
Supplement: Peer review history

# Peer review: Referee response for version 1

**DOI:** 10.21956/wellcomeopenres.13665.r32954

**Published:** 2018-05-23

**Authors:** Glyn Lewis

**Affiliations:** 1 Division of Psychiatry, University College London, London, UK

This is a valuable study of the psychometric properties of the Major Depression Inventory (MDI) in Kenya. The authors rightly pose the issue about the use of self-administered scales in sub-Saharan Africa. However, they could also cite the work of Vikram Patel in Zimbabwe who developed the Shona Symptom Questionnaire within the local population there. 

 The paper is very well written and easy to follow. It is clearly laid out and logical in its flow. The analysis is very thorough and seemed an appropriate approach towards studying the psychometric properties of the scale. 

 The main limitations of the article are primarily the absence of any comparison with other potential instruments. Furthermore, there is no comparison with a more detailed assessment to provide a more thorough "gold standard" measure. The arguments that given the prevalence is similar in Kenya to Denmark is not convincing. One of the main purposes of these scales is to compare the prevalence in populations and I would have thought these could well be different in Kenya and Denmark. 

 The further limitation is why they chose the MDI. They cite papers suggesting it performed better than some other competitors, and it has the attraction of brevity and I presume is not copyrighted. However, there are so many of these scales it seems rather arbitrary that they have chosen the MDI.  They state that the PHQ does not measure severity - but I don't think many people would agree with that. The PHQ is used as an outcome in the IAPT service in the UK and also in a number of clinical trials. I am not sure what they can do about this and they have tried to justify their choice in the intro.

I have read this submission. I believe that I have an appropriate level of expertise to confirm that it is of an acceptable scientific standard.

# Peer review: Referee response for version 1

**DOI:** 10.21956/wellcomeopenres.13665.r32130

**Published:** 2018-05-04

**Authors:** Sven Rabung, Kristina Abing

**Affiliations:** 1 Department of Psychology, Alpen-Adria-Universität Klagenfurt, Klagenfurt, Austria; 2 Department of Psychology, Alpen-Adria-Universität Klagenfurt, Klagenfurt, Germany

The authors report on the psychometric properties of the Major Depression Inventory (MDI) if administered in young people (aged 13-24 years) living in Coastal Kenya using a computer-assisted approach (Audio Computer-Assisted Self-Interviewing; ACASI) offering three different languages (Swahili, Giryama, and English). The results, although limited to feasibility, reliability in terms of internal consistency, and factorial validity, are in line with earlier reports on the psychometric properties of the MDI as determined in other cultural contexts thus speaking in favour of the MDI as being a suitable instrument for the assessment of depressive symptoms in rural Kenya.

 The present paper is exceptionally well written but could still be improved by some minor revisions:

1. In the Methods section it is stated that "Forward translation of the MDI to English and Giryama was done by one of the investigators". However, we assume that it should read "... to Swahili and Giryama ...".
2. In Table 1, the presentation of relative frequencies (%) line-by-line (instead of columnwise) appears somewhat counterintuitive.
3. In Table 2, the additional declaration of standard deviations of the MDI means would be helpful to assess the relevance of existing mean differences between subgroups.
4. In Table 3, item headings should be given in addition to item numbers for readers who are not familiar with the MDI.
5. Correspondingly, in Figure 1, item numbers should be added to the item headings.
6. In the paragraph "Prevalence of major depressive symptoms", the sentence "No statistically significant difference in the prevalence of DSM-IV major depressive symptoms was observed across sex and age sub-groups" seems to contradict the antecedent statement that "Female and older adolescents had higher prevalence of DSM-IV major depressive symptoms" as well as the subsequent results on categorical differences. 
7. here are some typos throughout the manuscript that should be corrected in the course of final copy editing.
8. Last not least, it would be of great importance to know where the Swahili and Giryama versions of the MDI can be obtained in order to promote their dissimination.

We have read this submission. We believe that we have an appropriate level of expertise to confirm that it is of an acceptable scientific standard.
